# Multi–predator Effects Produced by Functionally Distinct Species Vary with Prey Density

**DOI:** 10.1673/031.012.3001

**Published:** 2012-02-29

**Authors:** Ben P. Werling, David M. Lowenstein, Cory S. Straub, Claudio Gratton

**Affiliations:** ^1^ Department of Entomology, Michigan State University, East Lansing, MI, USA; ^2^ Department of Entomology, University of Wisconsin, Madison, WI, USA; ^3^ Biology Department, Ursinus College, Collegeville, PA, USA

**Keywords:** biodiversity, biological control, Colorado potato beetle, functional diversity, *Leptinotarsa decemlineata*, natural enemy, resource partitioning

## Abstract

Determining when multiple predator species provide better pest suppression than single species is a key step towards developing ecologically—informed biological control strategies. Theory and experiments predict that resource partitioning among functionally different predator species can strengthen prey suppression, because as a group they can access more prey types than functionally redundant predators. However, this prediction assumes that competition limits predation by functionally similar predators. Differences in prey density can alter the strength of competition, suggesting that prey abundance may modulate the effect of combining functionally diverse species. The experiment documented here examined the potential for functional differences among predator species to promote suppression of an insect pest, the Colorado potato beetle, *Leptinotarsa decemlineata* (Say) (Coleoptera: Chrysomelidae), at different prey densities. Predation was compared at two prey densities between microcosms that contained one predator species or two functionally distinct species: the lady beetle, *Coleomegilla maculata* De Geer (Coleoptera: Coccinellidae) that kills early *L*. *decemlineata* instars, and the soldier bug, *Podisus maculiventris* Say (Hemiptera: Pentatomidae) that kills late instars. The data show that combining these predators increased predation only when prey densities were low. This suggests that multiple predator species may only provide greater biological control than single species in systems where prey is limiting.

## Introduction

Determining when multiple predator species provide better pest suppression than single species is a key step towards developing ecologically—informed biological control strategies (Ehler 1990; Straub et al. 2008). Recent research suggests that resource partitioning among functionally distinct species can strengthen prey suppression, because functionally different predators can access more prey types than functionally redundant predators (Wilby and Thomas 2002; Pedersen and Mills 2004; Finke and Snyder 2008). Specifically, multiple predator species may consume more prey than single species when they forage for prey in different spatial locations or consume different prey stages or species (Wilby and Thomas 2002; Finke and Snyder 2008; Wilby et al. 2005; Straub and Snyder 2008). In the short term, such “functionally distinct” predators will be more efficient because they will be less likely to compete (Casula et al. 2006; Straub and Snyder 2008). Consequently, reduced competition could allow multiple species to coexist and consume more prey (Ives et al. 2005; Crowder et al. 2010).

Despite its importance, other ecological factors may determine whether positive effects of predator functional diversity are realized in multi-enemy communities. This study tested the hypothesis that prey density affects the relationship between predator functional diversity and predation. Specifically, it was predicted that predation by functionally similar and diverse predator communities would not differ when prey are abundant, because at high prey abundance competition should be low and should not limit predation rates, even for functionally similar predators (see Casula et al. 2006 for a similar discussion of predator abundance). Conversely, when prey is scarce, competition may be stronger (Yasuda et al. 2004), particularly among functionally similar predators (Casula et al. 2006). Thus, when prey is scarce, predation by functionally similar predators should be limited by competition, and communities with functionally diverse predators will consume more prey. These predictions were tested by combining two functionally distinct predators of *Leptinotarsa decemlineata* (Say) (Coleoptera: Chrysomelidae) at low and high densities of prey.

## Materials and Methods

Colorado potato beetle *Leptinotarsa decemlineata* feeds on potato foliage as a larva and adult (Hare 1990). *Leptinotarsa decemlineata* larvae molt three times after hatching from egg clusters: the first two instars, or “small larvae,” have an average wet mass of 1–3 mg, while third- and fourthinstars, or “large larvae,” weigh 27–105 mg (Matlock 2005). Initial trials suggested two common predators, the pink-spotted ladybird beetle *Coleomegilla maculata* De Geer (Coleoptera: Coccinellidae), and the twotwo-spinedspined stink bug *Podisus maculiventris* Say (Hemiptera: Pentatomidae), were functionally distinct in their predation of these life stages: *C*. *maculata* killed eggs and small larvae but not large larvae, while *P. maculiventris* killed large larvae but consumed fewer early instars (Werling 2009). A substitutive design was used to examine the effects of combining these functionally distinct predators while keeping abundance constant at two predators per cage (Straub and Snyder 2006). To determine if inferences about multi—predator effects vary with prey density, comparisons between polycultures and monocultures of these species were made at two densities of prey. Densities were based on per capita consumption rates in previous experiments (DML unpublished), and were expected to create a varying potential for competition. Specifically, the number of prey added to low and high density microcosms was approximately 75 and 300% of the consumption rates of the most effective predator of the relevant life stage (eggs and small larvae - *C*. *maculata*; large larvae - *P*. *maculiventris*). The resulting experiment was a factorial design with three combinations of predators (monoculture of *C*. *maculata*, monoculture of *P*. *maculiventris*, and polyculture) and two prey densities. Predation was measured in six replicates of each of the six combinations of predator diversity and prey density (*n =* 36). One replicate of the high—density *P. maculiventris* monoculture was discarded as only one predator was recovered at the end of the experiment, leaving a total of *n* —35.

Prey were exposed to predators in a greenhouse on one-month-old potted potato plants (*Solanum tuberosum* L. var. ‘Superior’) covered with 40 ×40 cm cloth sleeve cages (2 mm mesh size) supported by two bamboo stakes. Plants receiving a low density of prey were infested with an egg mass containing 10 eggs, four small larvae, and two large larvae, while high—density plants were infested with an egg mass containing 42 eggs, 18 small larvae, and six large larvae. Eggs on leaf discs were glued to undersides of leaves with water—soluble white glue. Predators were starved overnight before introduction to microcosms and removed after 48 hours, after which the number of prey remaining was counted. Predation was quantified as the proportion of each life stage killed averaged over the three life stages, AP, calculated as:


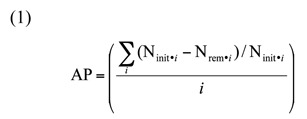


where *i* denotes the *i* ^th^ prey life stage, N_init_._i_ is the initial number of prey of the *ith *life stage, N_rem.i_ is the number of the *ith* life stage remaining after 48 hours, and there are *i* = 3 total life stages. Predation was measured in six replicates of each diversity x prey density combination, for a total çof 36. Note, some large larvae left plants and entered the soil to pupate during the experiment (24/124 larvae). These larvae were considered to have survived predation (i.e., were counted in Nrem.i). A two—way ANOVA (with prey density and predator combination as factors) suggested the proportion of larvae that pupated did not significantly vary between treatments (F_5_,_30_ = 0.53, *p=* 0.75). Given this, and the fact that larvae were randomly assigned to cages, it is unlikely that pupation of large larvae affected the outcome of the experiment.

**Figure 1.**
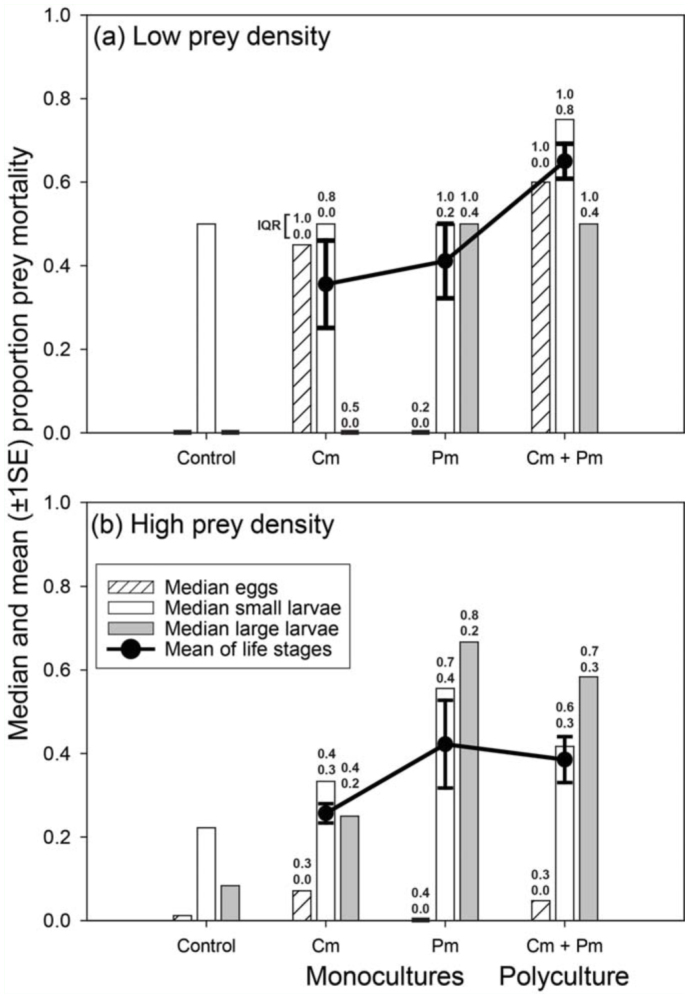
The median proportion of *Leptinotarsa decemlineata* eggs, small and large larvae killed, and the average proportion of all life stages that died (Equation 1 in Materials and Methods) in no-predator controls and in microcosms containing a monoculture of two individuals of *Coleomegilla maculata* (Cm) or *Podisus maculiventris* (Pm) and polycultures containing both species (Cm + Pm) at (a) low prey densities and (b) high prey densities. Numerals above bars indicate the upper and lower bound of the interquartile range (IQR) of proportional mortality of each life stage, while error bars show one standard error for the mean proportion of life stages that died. Zero medians are indicated by thickened lines on x-axis. Ranges (maximum minus minimum vales) of percent mortality for eggs, small and large larvae in controls were 0, 50, and 0% at low (n = 3) and 2, 0, and 17% at high prey density (n = 2), respectively. High quality figures are available online.

Mortality of prey was also monitored in *n* = 3 and *n* = 2 predator-free microcosms at low and high prey density, respectively. As expected, the average proportion of prey that died in the *n* = 35 microcosms with predators was significantly greater compared to controls, suggesting predators were consuming *L*. *decemlineata* (t—test assuming unequal variances: *t* = *5.7*, *p <* 0.01, d.f = 21). Controls were not included in further analyses due to low replication but are presented graphically for reference (Figure 1).

## Statistical analysis

A two-way permuted multivariate analysis of variance (PERMANOVA) (Anderson 2001) was used to determine if predation of multiple *L*. *decemlineata* life stages (eggs, small larvae, and large larvae) differed between monocultures of *C*. *maculata* and *P*. *maculiventris*, and if patterns changed with prey density. PERMANOVA is a non— parametric analog of MANOVA; it tests if multivariate variation between groups is larger than within—group variation. The Euclidean distance between each replicate in terms of the proportion of prey of each life stage killed was used to quantify multivariate variation in predation. The SIMPER module of PRIMER 6 (PRTMER-E Ltd. 2006) was then used to describe the percentage of the total multivariate difference between species that was due to differential predation of each prey life stage (Clarke and Gorley 2006).

The effects of functional diversity on predation were investigated using a two—way ANOVA with pre—planned contrasts implemented in R version 2.12.1 (R Core Development Team 2010). Two sets of pre— planned contrasts were used to test for multi— predator effects on predation. First, contrasts were used to test for differences in predation between the one— and two—species treatments at low and high prey density. A second set of contrasts were then conducted to discriminate between alternate explanations for greater predation in the two—species treatment. Specifically, the two—species treatment could have higher resource consumption than the one—species treatment because every replicate community contains the best performing species, compared to only one—half of the replicate communities in the one-species treatment (i.e., a sampling effect of diversity), and/or because functionally distinct predators partition resources in the two-species treatment, while functionally redundant predators compete for resources in the one species treatment (i.e., a species complementarity effect). Higher resource consumption in polycultures than the best performing monoculture provides unambiguous evidence for species complementarity (Tilman et al. 2001; Snyder et al. 2006). Thus, predation was also contrasted between the two-species treatment and best performing monoculture for both prey densities. The “gls” function of the “nlme” package of R (Pineheiro et al. 2010) was used to fit two-way ANOVA's with separate variance estimates for each combination of the two treatments, which accounted for heteroskedasticity and significantly improved model fit over the homogenous variance model (Likelihood ratio test: χ= 12.7, d.f = 5, *p <* 0.05). Contrasts, means, standard errors, and confidence intervals were obtained using the “contrast” package of R (Kuhn et al. 2010). Medians and interquartile ranges were calculated using IMP version 8.0.2 (SAS Institute 2009).

## Results and Discussion

In greenhouse microcosms, *C*. *maculata* and *P*. *maculiventris* differed in their relative consumption of different *L*. *decemlineata* life stages (Figure 1). Predation differed between monocultures of these two species (PERMANOVA Species effect: F1,19 = 4.14, *p <* 0.01), and there was no evidence this difference changed with prey density (Figure 1; Species x prey density: *F1,19* = 1.21, *p* = 0.31). The majority of this difference was due to *P*. *maculiventris* killing large larvae at a higher rate than *C*. *maculata* (40% of multivariate difference) and *C*. *maculata* killing more eggs (38% of difference; Figure 1). *Podisus maculiventris* also preyed on *L. decemlineata* small larvae at a slightly higher rate, although this contributed to differences between the two predators to a lesser degree (22%; Figure 1).

Combining these functionally distinct predators increased predation, but only when prey densities were low (Figure la). At low prey density, the average proportion of prey killed in polycultures was 83 and 58% greater compared to monocultures of *C*. *maculata* and *P*. *maculiventris*, respectively. Accordingly, mean predation in polycultures was significantly greater than the average of the two monocultures *(t* = 3.31, d.f = 29, *p<*0.01). Further, predation was greater in polyculture compared to the best performing monoculture, *P*. *maculiventris* (*t* = 2.42, d.f = 29, *p <* 0.05). This suggests the observed increase in predation was not produced simply because polycultures always contained the species that consumed the most prey (*P*. *maculiventris*), but instead was a result of resource partitioning by functionally distinct predator species. In contrast, there was little difference in predation between microcosms with one versus both of these species when prey densities were high (Figure lb; *p >* 0.55 for both contrasts at high density). These results are consistent with the hypothesis that competition limits predation by functionally similar predators at low, but not high, prey density. However, it is possible that multiple density—dependent mechanisms contributed to the results.

As expected, single-species predator communities killed fewer prey life stages than multi—species communities, but only when prey density was low. Other studies have found that differences in predator density can modify multi—predator effects. For example, Griffin et al. (2008) found that combining functionally distinct crab species led to greater prey consumption when predator densities were high, but not when they were low. Differences in aphid predation between predator polycultures and monocultures also increased with predator density (Griffiths et al. 2008). Taken together, the results of these three studies (Griffin et al. 2008; Griffiths et al. 2008; this study) suggest it may be the ratio of predators to prey, and not the absolute abundance of predators or prey, that matters. This is consistent with the importance of competition in driving positive multi—predator effects (Casula et al. 2006). Thus, multiple predator species may only provide better biological control than single species in systems where prey is limiting.

## **Abbreviations**

ANOVA,: analysis of variance;
AP: Average proportion of L. *decemlineata* life stages predated, defined by Equation 1 in the text;
PERMANOVA: permuted multivariate analysis of variance
